# Recovery of Acid and Alkaline from Industrial Saline Wastewater by Bipolar Membrane Electrodialysis under High-Chemical Oxygen Demand Concentration

**DOI:** 10.3390/molecules27217308

**Published:** 2022-10-27

**Authors:** Xiangfei Lü, Shuai Shao, Jinlong Wu, Yongguo Zhao, Bishuai Lu, Jieying Li, Linlin Liang, Lei Tian

**Affiliations:** 1 School of Water and Environment, Chang’an University, No. 126 Yanta Road, Xi’an 710054, China; 2 Shaanxi Research Design Institute of Petroleum and Chemical Industry, Xi’an 710054, China; 3 CCCC First Highway Consultants Co., Ltd., No. 63 Keji 2nd Road, Hi-Tech Development Zone, Xi’an 710075, China

**Keywords:** industrial saline wastewater, bipolar membrane, electrodialysis, chemical oxygen demand

## Abstract

Actual high saline wastewater containing concentrated organics and sodium chloride is a bioenergy and renewable resource. This study compared two different bipolar membrane electrodialysis membranes from two companies’ stacks to recover HCl and NaOH from sodium chloride solution and actual chemical wastewater. The results demonstrated that the electrolysis rates were around 1.5 kg/m^2^h, the HCl and NaOH production rates were about 0.9 kg/m^2^h, energy consumption was in the range of 1.05–1.27 kWh/kg, and the economic benefits were above 1 yuan/h in BMED systems. From analyzing the performance of seven different BMED membrane stacks, the B2 stack was chosen for electrolyzing actual high salt wastewater to observe the effect of chemical oxygen demand on BMED systems, where electrolytic salt performance, HCl-NaOH alkali production rates, and energy consumption show linear dependence on time for 5000 mg/L chemical oxygen demand wastewater. It illustrated chemical oxygen demand can enhance energy consumption and reduce electrolytic salt performance and the acid and alkali production rates, due to improving the membrane area resistance. In this study, the effect of high COD saline wastewater on the performance of a BMED membrane stack was clarified and the mechanism was analyzed for its practical application in treating chemical high salt wastewater.

## 1. Introduction

Highly saline wastewater is defined as wastewater containing organic matter and total dissolved solids (TDS) at a concentration of at least 3.5% by mass [1]. It mainly comes from production processes in several industries such as chemical, pharmaceutical, petroleum, paper, dairy processing, and food canning. Highly saline wastewater from chemical enterprises contains complex types of salts, which may contain various types of sodium salts, calcium salts, phosphates, etc. [2], and its salt content is high and can change from 5000 mg/L to 8000 mg/L. Since highly saline wastewater is produced by production plants of different chemicals, it leads to large water quality differences and water quantity fluctuations, and it contains organic matter, bacteria, ammonia nitrogen, inorganic salts, calcium, magnesium, barium, strontium, SiO_2_, fluorine, sulfate, and many other pollutants.

The disposal of saline water generated from the chemical industry has been gaining interest, due to its potentially detrimental effect on terrestrial and aquatic ecosystems [3,4]. Typically, the presence of high salinity in this waste stream makes it difficult to treat using a conventional biochemical system, because of the adverse effects of salt on microbial flora. Among all treatment technologies, bipolar membrane electrodialysis (BMED) has attracted more and more attention from researchers around the world, as an environmentally friendly technology [5,6]. Based on the ion-exchange membrane, BMED can split water into H^+^ and OH^–^ and then produce acid and base from the corresponding salt under a direct electric field. Furthermore, the conversion of salt into base and acid by BMED from wastewater containing a high salinity and organic matter gives a new avenue to raw material recycling and zero liquid discharge [7].

Therefore, since BMED was incipiently adapted to practical industrial applications in the 1980s, there has been a large number of experiments conducted using this technology to extract resources from high-salinity wastewater [8,9]. Mohammadi Mariam rapidly separated ammonia from high salt wastewater via BMED technology and recycled it into ammonium hydroxide solution [10]. Arif Hussain designed a multi-stage intermittent process in a BMED process which can further increase the concentration of acids and bases prepared via BMED technology [11]. BMED technology can also produce ultrapure deionized water and is used in the chemical industry for deep cleaning of organic and inorganic substances and producing organic and inorganic acids and bases from the respective salts [12,13,14,15]. The BMED process has been used in the production of organic acid, inorganic acid, and ammonia, from wastewater, to realize zero liquid discharge. Applying BMED, many researchers recovered NaOH and HCl from NaCl conversion in different wastewater [16,17,18,19]. However, the actual use of BMED technology for chemical high-salt wastewater treatment is still faced with the problem of easy contamination deactivation of the membrane stack during use. A large number of suspended solids (SS), scaling ions, and chemical oxygen demand (COD) in wastewater can easily enter the membrane pores and cause clogging, while the original membrane loses its decomposition performance [20]. Among them, SS can be controlled by microporous membrane filtration within 0.1 mg/L so that it does not affect the BMED membrane stack. Scaling ions can be engineered to be less than 1 mg/L by the sodium bed and chelate bed processes, thus eliminating their contamination of the membrane stack. An unresolved issue that has a significant impact on the operation of the BMED membrane stack is the COD in the wastewater. The COD in chemical wastewater is mainly composed of oxidizable organic molecules. Organic macromolecules carrying positive or negative electrical charges (i.e., polyelectrolytes, humic acids, surfactants, and dyes) tend to be preferentially deposited on the surface of the pore wall of ion exchange membranes, dramatically increasing the membrane resistance under a direct current.

It is significant to work to distinguish the effect of COD on bipolar membrane electrodialysis. Especially, how does the COD lead to the performance of BMED membrane stacks? In light of these considerations, this paper explores the specific effects of high COD concentrations on the acid and alkaline production performance of BMED membrane stacks to analyze the effect of COD on the acid and alkaline production performance of industrial saline wastewater. The new findings of this work are of considerable significance to study the pattern of COD affecting acid and alkaline energy production in BMED membrane stacks.

## 2. Results and Discussion

### 2.1. Comparative Analysis of Two Types of BMED Membrane Stacks

The core of the BMED system is the bipolar membranes. The system needs to be equipped with CEM and AEM and BMP. The properties and the orders of these membranes could affect the conversion of the salts into their corresponding acid and base [21,22]. Two types of CEM and AEM and BMP arranged in a different order were used to build different membrane stacks and numbered as A1, A2, A3, B1, B2, B3, and B4, respectively. The rate of electrolysis NaCl, HCl production, NaOH production, electrolysis NaCl power consumption per unit mass, and economics of acid-alkali production from electrolytic salt are used to analyze the performance of the BMED system.

#### 2.1.1. Comparative Analysis of Electrolytic Salt Rates of Different BMED Membrane Stacks

**Figure 1:**
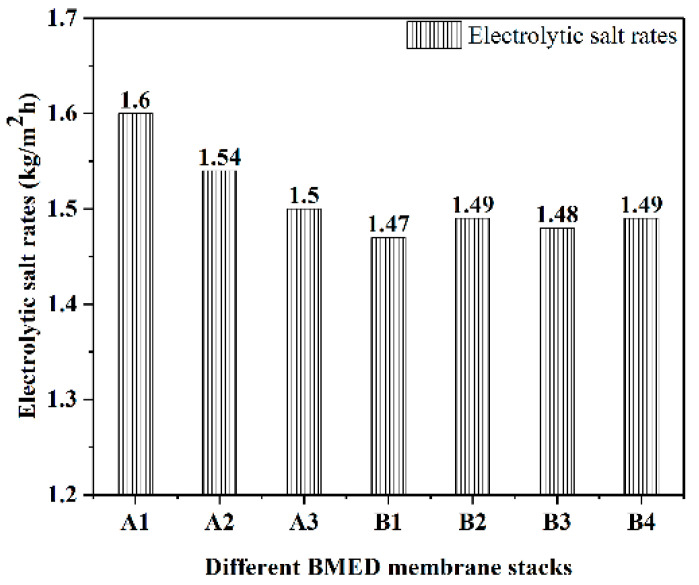
The electrolytic salt rates of different BMED membrane stacks.

In this work, it provides information about the electrolytic salt rates of different BMED membrane stacks at an initial concentration of 10% NaCl solution and a current density of 80 mA/cm^2^, shown in Figure 1. Overall, the electrolysis rates of all membrane stacks were around 1.5 kg/m^2^h, and the performance was stable, with an average of 1.5 kg of salt electrolyzed in 1 h on a 1 m^2^ membrane stack. Compared with some other experiments of NaCl removal via the BMED membrane stack, they exhibited an effective treatment for the salt in water. Compared with B1–B4, A1–A3 BMED stacks had a higher rate of electrolytic salt, but their stability was lower.

The electrolytic salt rates of A1–A3 BMED stacks were greater than 1.5 kg/m^2^h, while the electrolytic salt rates of B1–B4 BMED stacks were less than 1.5 kg/m^2^h. The average electrolytic salt rates of A1–A3 membrane stacks were 5% higher than those of B1–B4 membrane stacks. Although the electrolytic salt rate of the A1–A3 membrane stack is higher than that of the B1–B4 membrane stack, its stability is lower than that of the B1–B4 membrane stack. In actual engineering operation, if the difference in membrane stack performance is not large, its stability is more important for the treatment of high-salt wastewater. Since the membrane stack needs to be replaced when it is deactivated in experiments, it is important to ensure that the performance of the membrane stack does not change much after replacement as much as possible.

#### 2.1.2. Comparative Analysis of Acid and Alkali Production Rates of Different BMED Membrane Stacks

**Figure 2:**
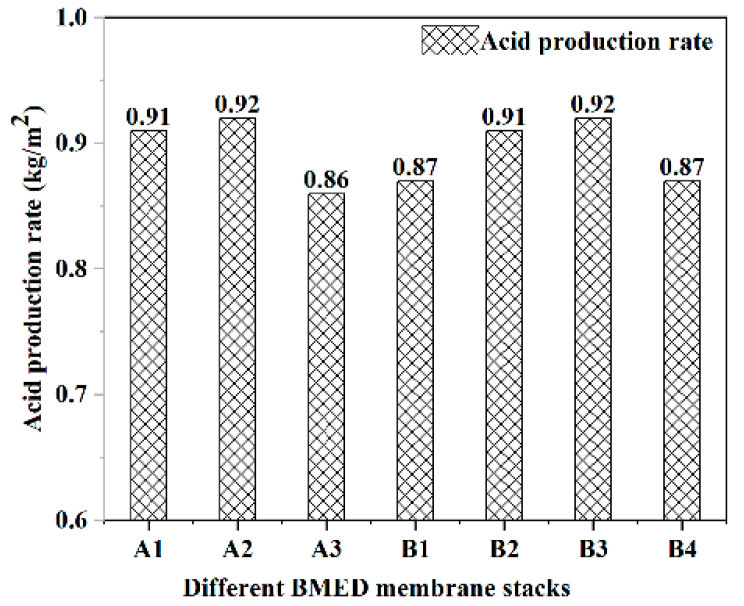
The acid production rates of different BMED membrane stacks.

**Figure 3:**
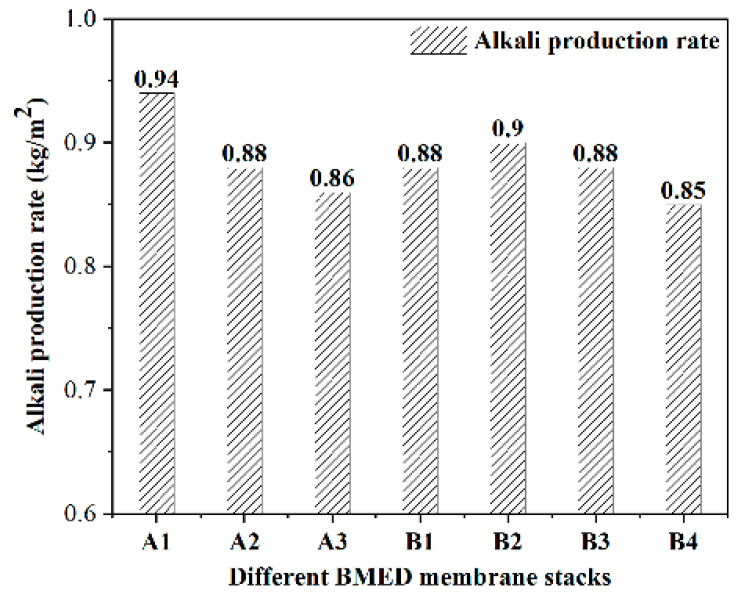
The alkali production rates of different BMED membrane stacks.

**Figure 4:**
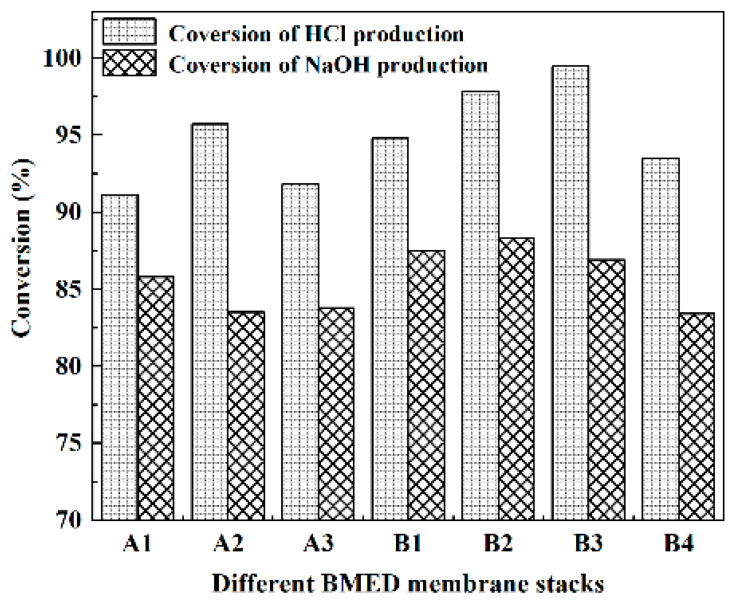
The conversion production rates of different BMED membrane stacks.

Figure 2 and Figure 3 represent the acid and alkali production rates of different BMED membrane stacks, respectively. Simultaneously, Equations (4) and (5) were used to calculate the conversion rate of acid and alkali production via electrolysis of salt with the BMED membrane stacks, shown in Figure 4. From an overall view, the acid and alkali production rates of all BMED membrane stacks were around 0.9 kg/m^2^h, and the stability of group B membrane stacks is stronger than group A membrane stacks. It shows that both BMED membrane stacks have higher acid conversion than alkali conversion for NaCl electrolysis. Nowadays, some membrane stacks in the market also have better performance in acid production than in alkali production [23]. In the performance of acid and alkali production, group B membrane stacks are slightly better than group A membrane stacks.

The highest acid production rate of the group A membrane stack (0.92 kg/m^2^h) is the same as that of the group B membrane stack (0.92 kg/m^2^h), but the lowest acid production rate of the group A membrane stack (0.86 kg/m^2^h) is lower than that of the group B membrane stack (0.87 kg/m^2^h). The highest alkali production rate of the group A membrane stack (0.94 kg/m^2^h) is higher than that of group B membrane stack (0.90 kg/m^2^h), but the lowest alkali production rate of group A membrane stack (0.86 kg/m^2^h) is lower than that of group B membrane stack (0.87 kg/m^2^h). The conversions of acid production from different BMED membrane stacks are above 91%, with the highest conversion being 99.5%. The conversion of alkali production from both BMED membrane stacks was above 83% with the highest being 88.3%. By comparing the acid and alkali production conversion rates of two types of BMED membrane stacks, it can be found that the acid and alkali production conversion rates of B group membrane stacks are higher, while the acid and alkali production rates are closer to each other.

#### 2.1.3. Comparative Analysis of Energy Consumption per Unit Mass of Different BMED Membrane Stacks

**Figure 5:**
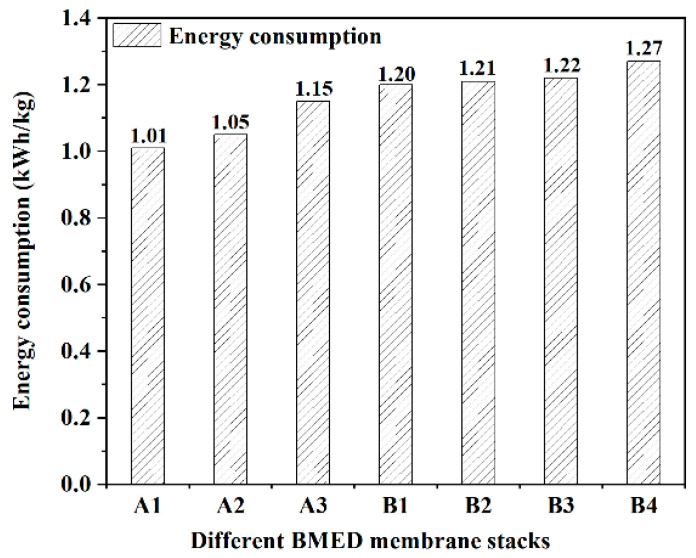
The energy consumption of different BMED membrane stacks.

Figure 5 shows the power consumption per unit of NaCl electrolyzed via seven membrane stacks at 10% NaCl brine. In general, the electrolytic salt unit power consumption of the type A membrane stack in this experiment is better than that of the type B membrane stack, and the cost of the type A membrane stack will be lower in practical application. The unit power consumption of electrolytic salt for the type A membrane stack is less than 1.15 kWh/kg, and the unit power consumption of electrolytic salt for the group B membrane stack is 1.2–1.3 kWh/kg.

#### 2.1.4. Comparative Analysis of the Economics of ACID-Alkali Production from Electrolytic Salt of Different BMED Membrane Stacks

**Figure 6:**
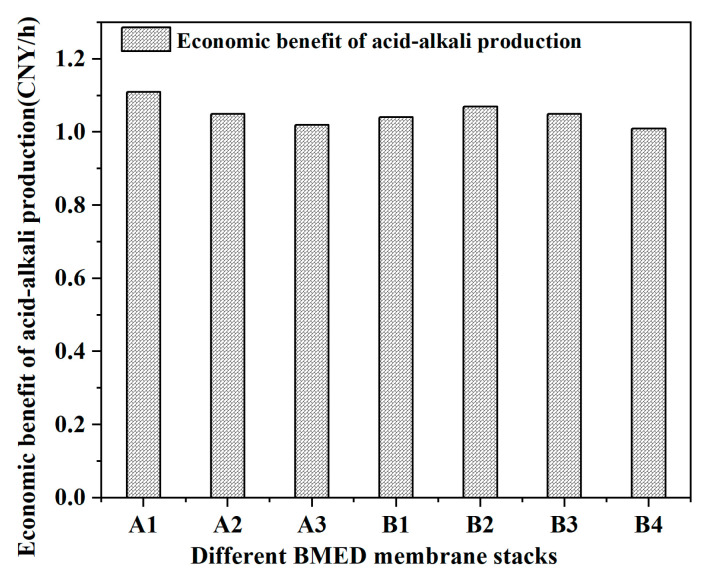
The economic benefit of acid-alkali production from different BMED membrane stacks.

According to the market price of the two kinds of acid and alkali, combined with their yields, Figure 6 represents the economic benefits of the two membrane stacks (the total value of the acid and alkali generated per square meter of BMED membrane stacks per hour). The economic benefits generated by each square meter of all BMED membrane stacks were above 1 yuan/h and the economic benefit of acid and alkali can reach 8000 yuan/year. Furthermore, the market price of sodium hydroxide in China is 922 Yuan/ton, while the price of hydrochloric acid in China is 262 Yuan/ton. Sodium hydroxide has a higher market price than hydrochloric acid. Therefore, if the BMED membrane stack produced more sodium hydroxide than the others, and its own membrane cost was lower, it is more suitable for practical application. Compared with group A membrane stacks, group B membrane stacks showed better economic benefits, due to a lower membrane cost and higher NaOH yield. Among all the BMED membrane stacks, B2 exhibited the most economic benefits, higher NaOH yield, excellent electrolysis, and low power consumption. In summary, B2 membrane stacks were chosen to electrolyze actual high-salt wastewater to observe the effect of COD on BMED systems.

### 2.2. Effect of High COD Saline Wastewater on the Performance of BMED Membrane Stacks

Combined with the stability of the electrolytic salt rate and the conversion rate of acid, the base production of the group B membrane stack obtained in the previous section is better than the type A membrane stack, and the cost of type B membrane stacks is lower than type A membrane stacks over the life of the BMED membrane stack in the actual production process. Therefore, it is more appropriate to choose a group B membrane stack for an engineering operation, and a group B membrane stack is also used in this experiment.

#### 2.2.1. Effect of High COD Saline Wastewater on Electrolytic Salt Performance

**Figure 7:**
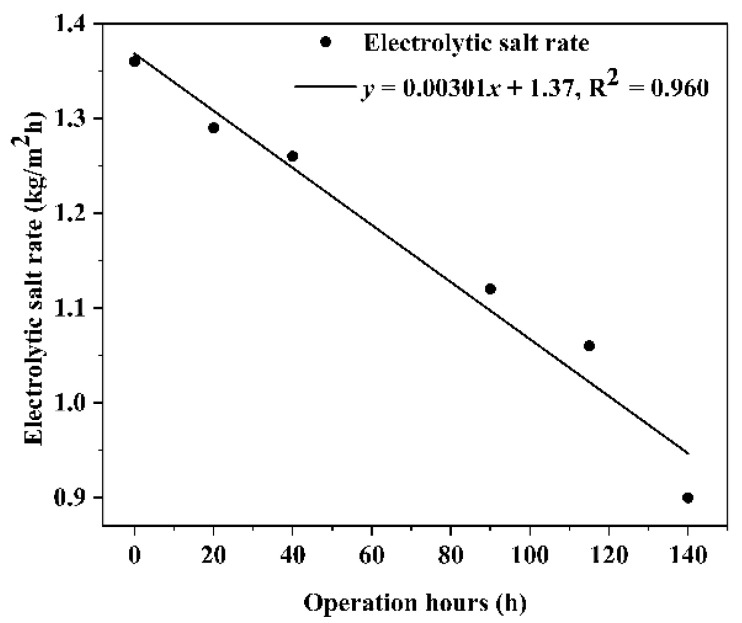
The electrolytic salt rates of high COD saline wastewater by BMED membrane stacks.

The electrolytic salt rates were calculated for BMED membrane stack operation at 0 h, 20 h, 40 h, 90 h, 115 h, and 140 h under the operating conditions of concentrated brine with COD of 5000 mg/L, and the results are shown in Figure 7. As seen in the figure, the electrolytic salt rate of the BMED membrane stack decreases faster as the operation time goes on. It indicates that the membrane stack is contaminated with COD under the operating conditions of high COD concentrated brine. The electrolytic salt rate of the membrane stack decreases faster as the operation time goes on, which indicates that the membrane stack is being contaminated by COD. The electrolytic salt rate of the membrane stack decreased from 1.36 kg/m^2^h at the beginning to 0.90 kg/m^2^h after 140 h of operation, and the electrolytic salt rate of the membrane stack decreased by 33%, illustrating that the COD contamination had a large impact on the electrolytic salt rate of the membrane stack.

#### 2.2.2. Effect of High COD Saline Wastewater on the Acid and Alkali Production Rates of Industrial High Saline Wastewater

**Figure 8:**
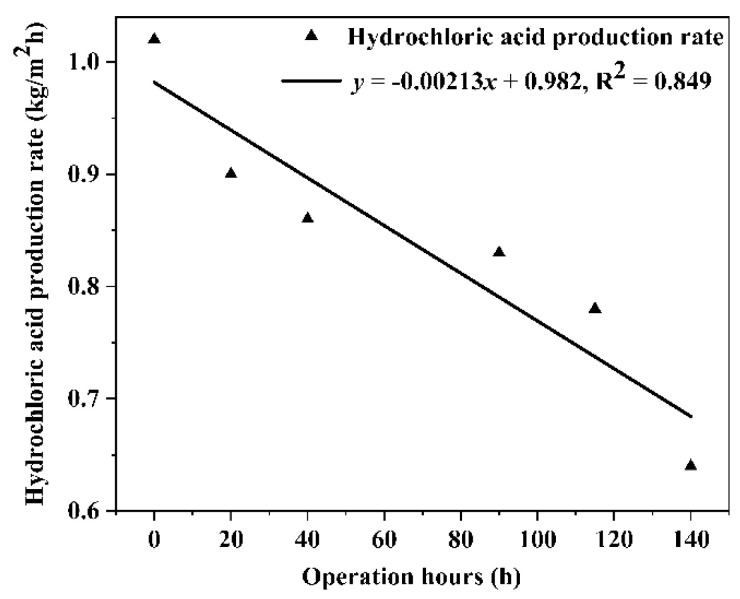
The variation of acid production from high COD saline wastewater via BMED membrane stacks.

**Figure 9:**
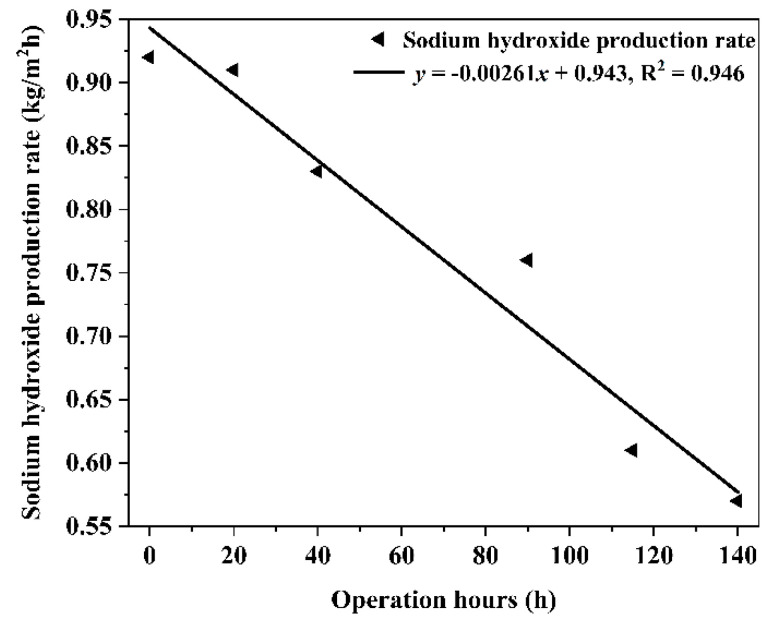
The variation of alkali production from high COD saline wastewater via BMED membrane stacks.

Under the operating conditions of concentrated brine with COD of 5000 mg/L, the acid and alkali production rates of electrolytic salt were calculated for BMED membrane stack operation at 0 h, 20 h, 40 h, 90 h, 115 h, and 140 h. The results are shown in Figure 8 and Figure 9, respectively. As shown in the figure, the rates of acid and alkali production from the electrolytic salt of the BMED membrane stack are decreasing. It exhibited that the variation of HCl and NaOH production rates of saline wastewater via the BMED system followed a similar downward trend. After operating continuously for 140 h, the HCl production rate of the BMED membrane stack decreased from 1.02 kg/m^2^h to 0.64 kg/m^2^h, down 37%, while the NaOH production rate of the BMED membrane stack decreased from 0.92 kg/m^2^h to 0.57 kg/m^2^h, down 38%. This indicated that the BMED membrane stacks were contaminated with COD under the operating conditions of high COD concentrated brine.

#### 2.2.3. Effect of High COD Saline Wastewater on Energy Consumption Per Unit Mass of BMED System

**Figure 10:**
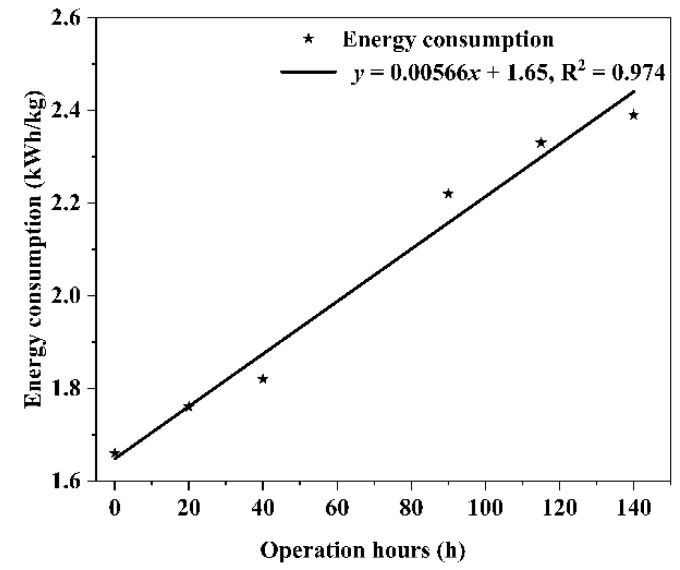
The energy consumption of high COD saline wastewater via BMED membrane stacks.

In the initial chemical oxygen demand (COD) concentrations of 5000 mg/L saline wastewater, the energy consumption per unit mass of the BMED system during continuous operation for 140 h was calculated using Equation (7). The result is shown in Figure 10. It was found that the energy consumption per unit mass of the BMED system continued to rise with the increase of operating time, due to the contamination of COD. After operating continuously for 140 h, the electrolytic salt unit power consumption increased from 1.66 kWh/kg to 2.39 kWh/kg, a growth of 44%. This indicated that the resistance of the membrane stack was increasing in the BMED system.

### 2.3. The Possible Mechanism Analysis

**Figure 11:**
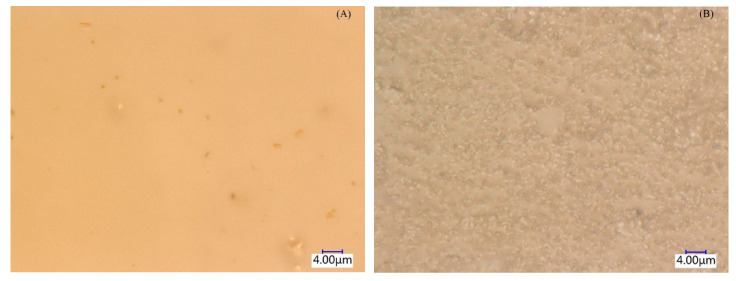
The extended depth-of-field microscopic images between new bipolar membrane (**A**) and used bipolar membrane (**B**).

According to the literature [24], whether high saline wastewater is suitable for BMED treatment mainly depends on its energy consumption, which is related to the resistance of the BMED system. In this work, 5000 mg/L COD illustrated there are high concentrations of organic pollutants in saline wastewater, which is a significant limitation for the BMED process, due to the fouling on the membrane surface. Especially, the organic pollutants in industrial saline wastewater are easily adsorbed on the surface of BMED membranes, which is the gallery between the membrane functional group and solutions, and then increase the membrane area resistance. The extended depth-of-field microscopic images between the new bipolar membrane and used bipolar membrane in 5000 mg/L COD wastewater are exhibited in Figure 11. The surface of the new bipolar membrane is very clean and smooth, while the surface of the used bipolar membrane in 5000 mg/L COD wastewater is contaminated by pollutants. To a certain extent, the results in Section 3.2 reflect the organic pollutants accumulated on the surface of the membrane over time during the experiment.

## 3. Material and Method

### 3.1. Materials

The chemicals, such as NaCl and AgNO_3_, are analytical purity grades, while NaOH and HCl are superior purity grades. They were purchased from a domestic chemical reagent company. All solvents and reagents were used as received without further purification. Distilled water was used throughout. The ion-exchange membranes of the BMED system were provided by Hangzhou Cree Environmental Energy Technology Co., Ltd., Hang Zhou, China. The main properties of the BMED membranes used in this study are listed in Table 1.

### 3.2. Experiments

#### 3.2.1. BMED Membrane Stacks under Standard Brine

**Figure 12:**
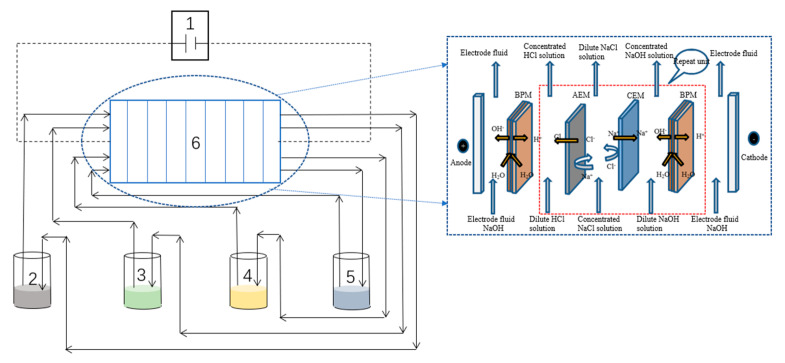
Schematic diagram of the integrated setup. (1) DC power supply; (2) electrode storage tank; (3) alkali storage tank; (4) acid storage tank; (5) salt storage tank; (6) BMED membrane stack.

Figure 12 shows the integrated experimental setup, which comprises a (1) DC power supply (rated voltage 35 V, rated current 4.4 A), (2) electrode storage tank, (3) alkali storage tank, (4) acid storage tank, (5) salt storage tank, and (6) BMED membrane stack. The BMED membrane stack consists of ten repeated units, which contained two pieces of bipolar membrane, one piece of anion exchange membrane, and one piece of cation exchange membrane, constituting two electrode storage tanks, one acid storage tank, one alkali storage tank, and one salt storage tank. The tanks were separated by anion/bipolar membranes, plastic partition sheets, and silicone gaskets. The single membrane size is 75 mm × 195 mm, and the effective area of the membrane stack is 0.055 m^2^. The electrodes were made of titanium coated with ruthenium.

In this experiment, two types of membrane stacks were tested and numbered as A1, A2, A3, B1, B2, B3, and B4, respectively, according to the different membrane stacks in Table 2. While A represented three groups in type A, B exhibited four groups in type B. NaOH solution (1 L, 4% wt) was added to the electrode storage tank (2). Both acid solution tank (4) and the alkali storage tank were filled with dilute water. According to the literature [21,22], NaCl solution (1 L, 10% wt) was added to salt storage tank (5) to confirm the BMEP system parameters. High salt content wastewater from chemical production wastewater was added to salt storage tank (5). According to previous work [25], the constant current density was set to 80 mA/cm^2^. Before starting the experiment, the dilute water was added to four storage tanks and circulated for 10 min to discharge air bubbles in the devices, which could reduce the influence of reducing the effective membrane area, due to the adsorption of air bubbles on the surface of the membrane. During the operation, the BMED device was turned on for 1 h and then disconnected from the DC power supply, and the pumps of each storage tank were kept on for 10 min and then turned off. The solutions in the salt, alkali, and acid storage tank were collected and analyzed at predetermined time intervals. Standard HCl solution (0.1 mol/L) was used for acid-base titration to determine the amount of NaOH from the alkali storage tank. Then, the standard sodium hydroxide solution (0.1 mol/L) was selected to confirm the amount of HCl generated in the acid storage tank. The AgNO_3_-based titration method was used to determine the content of Cl^−^ in the salt storage tank and further to calculate the concentration of NaCl solution in the salt storage tank. Each experiment was repeated three times. After the operation was stopped and other membrane stacks were replaced. All the experiments were performed according to the same conditions.

#### 3.2.2. The BMED Treatment of Salinity Wastewater under High COD Concentration

According to the literature [26,27], BMED systems are easily polluted by suspended solids, scaling ions, and COD in wastewater, resulting in an obvious decrease in the acid-base production from the membrane stacks. At present, the solid suspended solids and scaling ions in the wastewater are solved via the engineering process, but it is difficult to completely remove COD in high salinity wastewater. V. Lindstrand in Lund University (Sweden) found that organic molecules in wastewater caused organic fouling of ED anion exchange membranes in BMED stacks, resulting in increased membrane resistance, reduced ion transport through the membrane, and organic fouling increasing with time [28]. Among the above three factors, COD is the key factor affecting the service life of BMED, especially the high concentration of COD in actual chemical wastewater. Therefore, the effect of high COD on the performance of the BMED system could help us find an alternative and cost-effective strategy for treating high salinity wastewater.

**Figure 13:**
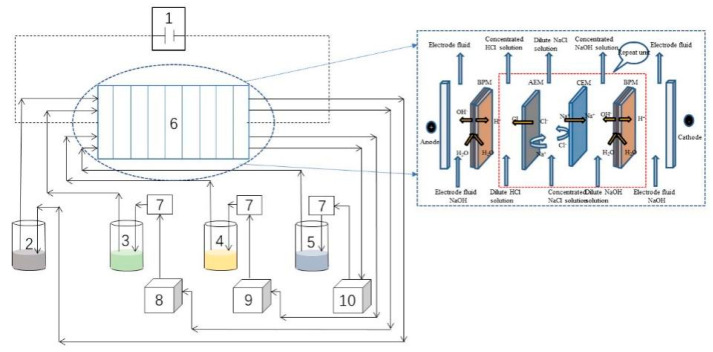
Schematic diagram of the integrated setup under 5000 mg/L COD. (1) DC power supply; (2) electrode storage tank; (3) alkali storage tank; (4) acid storage tank; (5) salt storage tank; (6) BMED membrane stack; (7) connectors; (8) external alkali storage tank; (9) external acid storage tank; (10) external salt storage tank.

In this experiment, the samples were selected from the chemical wastewater produced by a chemical plant in Yulin, Shaanxi Province, as the raw material. The high-salinity wastewater contains more than 10% salt content, and there is a large amount of SS, scaling ions, and COD in the brine. The SS and scaling ions in the high-salinity wastewater were removed before this experiment. The measured value of COD in the high salinity wastewater is 5000 mg/L. Due to the long-running time and large volume amount of actual wastewater, three external tanks with a volume of 20 L were linked to the alkali storage tank, acid storage tank, and salt storage tank, respectively (shown in Figure 13). This added tank can ensure the continuous and stable operation of the system for more than 20 h. When the experiment started, 20 L high salinity wastewater with 5000 mg/L COD was added to the salt storage tank, and 20 L distilled water was added to the acid storage tank and alkali storage tank, respectively. Then, 1 L 4% NaOH solution was added to the electrode storage tank. The BMED unit was turned on and operated steadily for more than 20 h. After that, the power supply was disconnected. The external salt, acid, and alkali tanks were disconnected. Finally, the external storage tank was connected again, and the salt storage tank was replaced with saline wastewater with a COD of 5000 mg/L and continued to run for more than 20 h. The experiment was completed in five cycles. The BMED membrane stack performance was tested at 20 h, 40 h, 90 h, 115 h, and 140 h, respectively. The influence of COD pollution on the performance of the BMED system was studied from three aspects: the rate of electrolysis NaCl, HCl production, NaOH production, and electrolysis NaCl power consumption per unit mass. These parameters were obtained via the analysis method in Section 2.3.

### 3.3. Data Analysis

The rate of electrolysis NaCl, HCl production, NaOH production, and electrolysis NaCl power consumption per unit mass were selected as process performance criteria for the BMED system, and other membrane stack parameters (economics of acid-alkali production from electrolytic salt, acid-alkali conversion rate) were also introduced to evaluate the membrane stack performance [8,23]. Experimental data were collected during the experiments and analyzed using the following equations.

The BMED membrane stack electrolysis rate of NaCl is calculated from Equation (1) as:

(1) $$v_{y}=\frac{\left(c_{y0}V_{y0}-c_{yt}V_{yt}\right)}{At}$$

Here, $v_{y}$ is the electrolysis NaCl rate (kg/ m^2^h), $c_{y0}$ is the initial NaCl concentration of the salt storage tank (mol/L), $V_{y0}$ is the initial NaCl solution volume of the salt storage tank, $c_{yt}$ is the NaCl concentration of the salt storage tank at time t (mol/L), $V_{yt}$ is the NaCl solution volume of the salt storage tank at time t, *A* is the effective membrane area (m^2^), and t is the running time (h).

The rate of HCl production from the BMED membrane stack was calculated from Equation (2):

(2) $$v_{s}=\frac{C_{s}\times M_{s}\times L_{s}}{At}$$

Here, $v_{s}$ is the rate of HCl production (kg/ m^2^h), $C_{s}$ is the concentration of HCl production (mol/L), $M_{s}$ is the molar mass of HCl (g/mol), $L_{s}$ is the volume of HCl solution (L), A is the effective membrane area (m^2^), and *t* is the running time (h).

The rate of NaOH production from the BMED membrane stack was calculated from Equation (3):

(3) $$v_{j}=\frac{c_{j}\times M_{j}\times L_{j}}{At}$$

Here, $v_{j}$ is the rate of NaOH production (kg/m^2^h), $c_{j}$ is the concentration of NaOH production (mol/L), $M_{j}$ is the molar mass of NaOH (g/mol), $L_{j}$ is the volume of NaOH solution (L), *A* is the effective membrane area (m^2^), and *t* is the running time (h).

The conversion of HCl produced by the BMED membrane stack is given using Equation (4):

(4) $$\gamma_{s}=\frac{c_{s}\times M_{s}\times L_{s}}{c_{y0}V_{y0}-c_{yt}V_{yt}}$$

Here, $\gamma_{s}$ is the conversion rate of HCl produced via electrolysis of NaCl in the BMED membrane stack, $c_{s}$ is the concentration of HCl produced (mol/L), $M_{s}$ is the molar mass of HCl (g/mol), $L_{s}$ is the volume of HCl-producing solution (L), $c_{y0}$ is the initial NaCl concentration in the salt storage tank (mol/L), $V_{y0}$ is the initial volume of NaCl solution in the salt storage tank (L), $c_{yt}$ is the NaCl concentration in the salt tank at moment *t* (mol/L), and $V_{yt}$ is the volume of NaCl solution in the salt storage tank at moment t (L).

The conversion of NaOH produced by the BMED membrane stack is given using Equation (5):

(5) $$\gamma_{j}=\frac{c_{j}\times M_{j}\times L_{j}}{c_{y0}V_{y0}-c_{yt}V_{yt}}$$

Here, $\gamma_{j}$ is the conversion rate of NaOH produced via electrolysis of NaCl in the BMED membrane stack, $c_{j}$ is the concentration of NaOH produced (mol/L), $M_{j}$ is the molar mass of NaOH (g/mol), $L_{j}$ is the volume of NaOH solution produced (L), $c_{y0}$ is the initial NaCl concentration in the salt storage tank (mol/L), $V_{y0}$ is the initial NaCl solution volume in the salt storage tank (L), $c_{yt}$ is the NaCl concentration in the salt tank at moment *t* (mol/L), and $V_{yt}$ is the NaCl solution volume in the salt storage tank at the moment *t*.

The economic efficiency of HCl and NaOH production from the BMED membrane stack is given using Equation (6):

(6) $$P=m_{s}\times s_{s}+m_{j}\times s_{j}$$

Here, *P* is the economic benefits of BMED membrane stack production of HCl, NaOH (yuan), $m_{s}$, $m_{j}$ are the amount of HCl, NaOH production (kg), and $s_{s}$, $s_{j}$ are the unit price of HCl, NaOH (Yuan/kg). 

The energy consumption per unit of NaCl electrolysis using the BMED membrane stack is given via Equation (7):

(7) $$w_{e}=\frac{I\sum U\cdot \Delta t}{m_{y}}$$

Here, $w_{e}$ is the energy consumption per unit of NaCl electrolysis (kWh/kg), *I* is the current of the membrane stack operation (A), *U* is the membrane stack voltage (V), *t* is the time to run the membrane stack (h), and $m_{y}$ is the amount of sodium chloride electrolysis (kg).

## 4. Conclusions

The application feasibility of the BMED system in high saline wastewater treatment was proved in this study. The BMED system was optimized for the electrolysis of sodium chloride solution and the production of HCl and NaOH. Different BMED membrane stacks were compared by electrolytic salt rates, acid and alkali production rates, energy consumption, and the economics of acid-alkali production. The results indicated that the electrolysis rates were around 1.5 kg/m^2^h, the HCl and NaOH production rates were about 0.9 kg/m^2^h, energy consumption was in the range of 1.05–1.27 kWh/kg, and the economic benefits were above 1 yuan/h. Among these BMED membrane stacks, the B2 membrane stack was chosen for electrolyzing actual high-salt wastewater to observe the effect of COD on BMED systems. During the recovery processing, the effect of 5000 mg/L COD on electrolytic salt performance, the acid and alkali production rates, and energy consumption were analyzed over time. The results indicated that the electrolytic salt performance, electrolytic acid production, and alkaline production decreased by 33%, 37%, and 38%, respectively, while the electrolytic salt power consumption increased by 44%. It was found that as the performance of the membrane stack increased with the time of contamination, the amount of COD contaminated by the membrane stack also increased, which caused a linear decrease in the electrolytic salt performance of the membrane stack, a linear decrease in the electrolytic acid and alkaline production rates with the contamination of the membrane stack, and an increase in the electrolytic salt power consumption. The influence of COD shows an obvious linear relationship with time. It was concluded that the BMED is a feasible and environmental process for the application of high saline wastewater recovery, before removing pollution from high concentrations of COD in wastewater.

## Figures and Tables

**Table 1:** Main properties of the ion exchange membranes used in the BMED system.

Membrane Type	Thickness (mm)	Blasting Strength (MPa)	Resistance (Ω·cm^2^)	Operating pH	Washing pH	Operating Temperature (°C)
BPM (NEOSEPTA)	0.22	≥0.4	-	0–14	0–14	≤40
BPM(LANRAN-BP-2)	0.28	≥1.0	-	0–14	0–14	25–40
CEM (Type 1)	0.21	≥0.4	4.5	0–14	0–14	≤60
CEM (Type 2)	0.28	>0.5	<8.0	0–14	0–14	25–60
AEM (Type 1)	0.11	≥0.15	2.6	0–8	0–8	≤40
AEM (Type 2)	<0.21	>0.5	<3.8	0–14	0–14	25–60

The data were provided from the relative company.

**Table 2:** Main properties of the ion exchange membranes used in the BMED system.

Membrane Type	Composition of the Membrane Stack		
	AEM	CEM	BPM
A1	Type 1	Type 1	NEOSEPTA Membrane
A2	Type 2	Type 2	NEOSEPTA Membrane
A3	Type 1	Type 2	NEOSEPTA Membrane
B1	Type 1	Type 1	NEOSEPTA Membrane
B2	Type 2	Type 2	NEOSEPTA Membrane
B3	Type 1	Type 1	LANRAN-BP-2
B4	Type 2	Type 2	LANRAN-BP-2

## Data Availability

All data were exhibited in figures and original data will be submitted to *Molecules*.

## Sample Availability

Samples of the compounds are available from the authors.
